# Impact of peripapillary staphylomas on the vascular and structural characteristics in myopic eyes: a propensity score matching analysis

**DOI:** 10.1007/s00417-022-05966-2

**Published:** 2023-01-09

**Authors:** Fen Nie, Lurong Zhang, Mengdan Cao, Dengming Zhou, Ke Liu, Junyi Ouyang, Lijia Luo, Ruiling Zhu, Shaosan Liu, Xuanchu Duan

**Affiliations:** 1 Department of Ophthalmology, The Second Xiangya Hospital, Central South University, Changsha, Hunan Province China; 2 Aier Glaucoma Research Institute, Changsha Aier Eye Hospital, Changsha, Hunan China; 3 Clinical Nursing Teaching and Research Section, The Second XiangYa Hospital, Central South University, Changsha, Hunan Province China

**Keywords:** Peripapillary staphylomas, Myopia, Vessel, Structure, Optical coherence tomography

## Abstract

**Purpose:**

To apply propensity score matching to evaluate the impact of peripapillary staphylomas (PPS) on vascular and structural characteristics in the myopic eyes.

**Methods:**

This was a prospective, cross-sectional study. Forty-one control eyes and 41 eyes with PPS were analyzed. The eyes were selected using propensity score matching analysis based on the age and axial length. All subjects underwent ophthalmologic examinations for assessing vessel and structure parameters using swept-source optical coherence tomography (SS-OCT), OCT angiography, color fundus photography, and ocular biometry.

**Results:**

As compared with control eyes, the eyes with PPS had shallower anterior chamber depth (3.61 ± 0.24 mm vs 3.77 ± 0.24 mm, *P* = 0.004), higher intraocular pressure (IOP) (16.59 ± 2.88 mmHg vs 14.53 ± 2.45 mmHg, *P* = 0.002), and higher myopic spherical equivalent (− 11.52 ± 3.22D vs − 9.88 ± 2.20D, *P* = 0.009). while corneal curvature and lens thickness between the two groups were not statistically different. Compared with control eyes, increased macular deep vessel density, reduced macular choriocapillaris and radial peripapillary capillary, and thinning retinal layer, ganglion cell complex, choroidal layer as well as the superior and inferior peripapillary retinal nerve fiber layer were observed in eyes with PPS, apart from larger disc area, parapapillary atrophy area, and degree of disc rotation. Logistic regression analysis revealed that the IOP (*P* = 0.046), disc rotation (*P* = 0.003), and average peripapillary choroidal thickness (*P* = 0.009) were associated with the presence of PPS.

**Conclusion:**

Close association of PPS with exacerbation of myopia and anatomical alterations was observed which not only affected the eye posterior segment but also the anterior segments. We further identified significant reductions in the radial peripapillary capillary and macular choroidal perfusion with the increase in macular deep retinal flow blood of myopic eyes with PPS. Higher IOP, thinner peripapillary choroidal thickness, and rotated optic disc were risk factors for the presence of PPS.

**Supplementary information:**

The online version contains supplementary material available at 10.1007/s00417-022-05966-2.


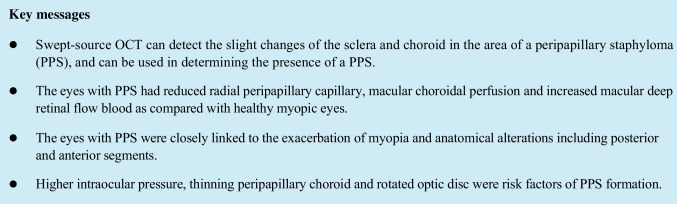


## Introduction

Posterior staphyloma (PS) is a hallmark of pathologic myopic eyes, which are classified into ten types, based solely on the ophthalmoscopic examination by Curtin [1]. However, PS were further reclassified by Ohno-Matsui into six types viz., wide macular PS, narrow macular PS, peripapillary PS (PPS), nasal PS, inferior PS, and others [2]. This classification was based on the contour of the outermost border of PS using ultra-wide-field fundus imaging system combined with three-dimensional magnetic resonance imaging. Curtin and Ohno-Matsui have reported the incidence of PPS around 1.5% and 5% respectively from all types of PS [1, 2]. However, it is of noteworthy that the prevalence of PPS remains extremely low.

Studies have confirmed that eyes with PS are significantly associated with the worsening of visual acuity and serious anatomical alterations than the non-staphylomatous eyes [3–5]. However, few comprehensive and quantitative studies have reported the specific effects of each type of PS on visual outcome and anatomical structure in highly myopic eyes. Particularly, probably due to low incidence of PPS, no study has identified its risk of damaging the macular and optic nerve.

Our previous research found that myopic PS is linked to lens thickening and anterior chamber depth (ACD) shallowing [3], then, whether PPS is also associated with anatomical abnormalities in the anterior segment? In a study involving 60 staphylomatous eyes with normal tension glaucoma, the number of eyes with PS involving the disc (*n* = 39), namely PPS, were significantly more than eyes where PS was engaged at the temporal side of the disc (*n* = 21) [6]. However, it was contradictory for the myopic staphylostoma. The result of this study pose question of the myopic eye with PPS is involved in the higher susceptibility of developing glaucomatous optic disc alterations than the other myopic eyes or glaucoma is one of the causes of PPS formation.

 The profound penetration advantage of swept-source optical coherence tomography (SS-OCT) has allowed ophthalmologists to examine the fundus structure in more detail [7–9]. With SS-OCT, deep tissues structures, such as the choroid, sclera, retrobulbar subarachnoid space, can be imaged with more precision [7–9]. Additionally, OCT angiography is a helpful tool for quantitative and noninvasive evaluation of the retinal and choriocapillaris microcirculation in vivo [10–12]. A study by Shinohara et al. has demonstrated that the SS-OCT examination is important to diagnose the presence of PPS [13]. Therefore, the current study was conducted to quantitatively evaluate the PPS probable association with the vascular and structural alterations in myopic eyes using SS-OCT and OCT angiography. Factors contributing to being PPS was also investigated.

## Subjects and methods

**Fig. 1:**
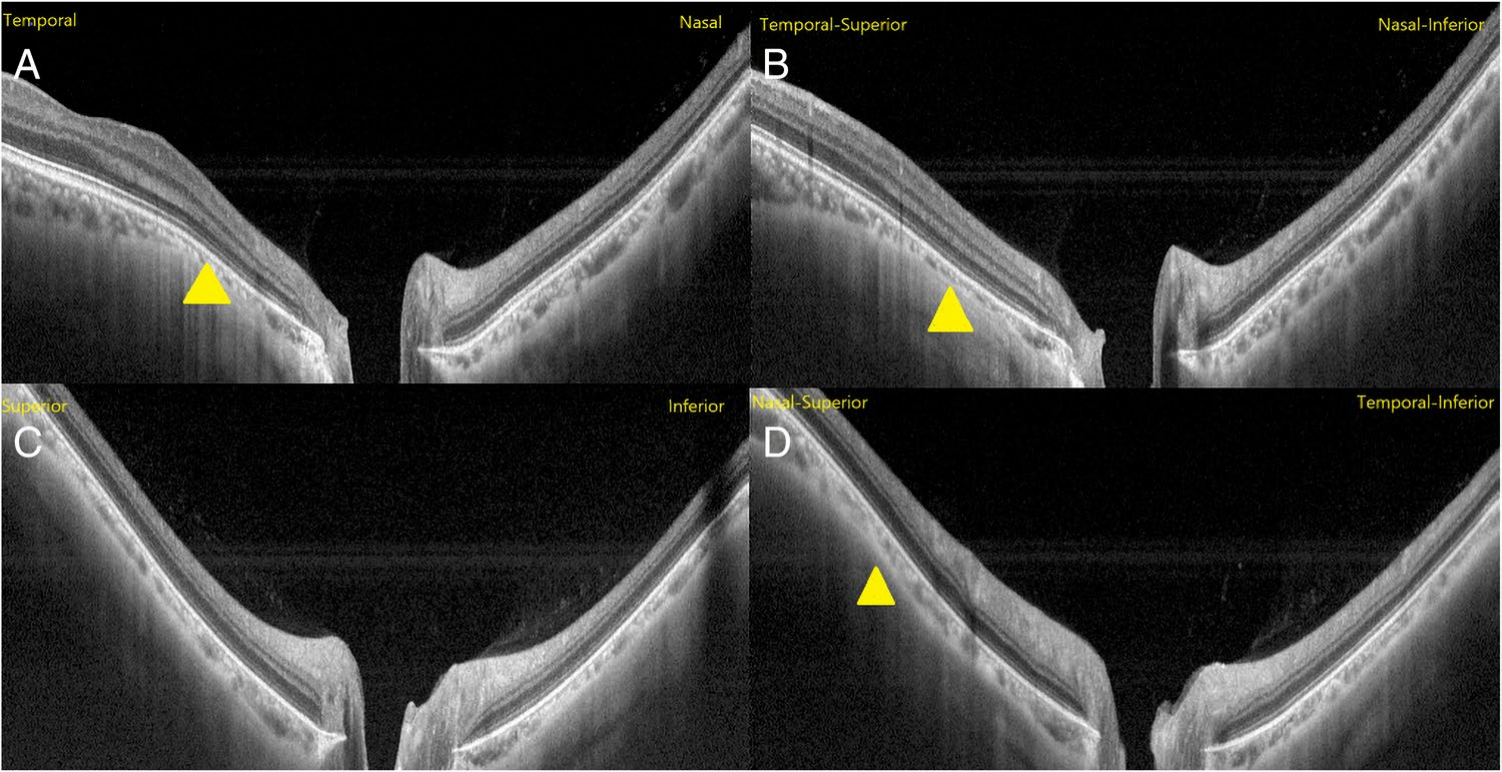
Images of PPS SS-OCT radial B-scans centered on optic disc. Image **A**: horizontal scan; Image **B**: temporal-superior to nasal-inferior scan; Image **C**: vertical scan; Image **D**: nasal-superior to temporal-inferior scan. The characteristic of an inward protrusion of the sclera compressing and thinning choroid at the staphyloma edge were observed in temporal, temporal-superior and nasal-superior (yellow triangle). The posterior sclera in the region of the PPS was bended posteriorly

This prospective, cross-sectional study adhered to the guidelines of the Declaration of Helsinki and was approved by the Ethics Committee of Changsha Aier Eye Hospital. Informed consent was obtained from each of the patients prior to the study. The study recruited 57 consecutive subjects with PPS and 401 control subjects with myopia without PS, who visited the Refractive Center of Changsha Aier Eye Hospital from September 2020 to August 2021. Each patient underwent a detailed ophthalmologic examination, including a medical history review, best-corrected visual acuity (BCVA), non-contact intraocular pressure (IOP), central corneal thickness (CCT), refraction, slit-lamp biomicroscopy, mydriatic indirect ophthalmoscopy, color fundus photography, SS-OCT (DRI OCT-1; Topcon Corp, Software Version 1.28.17642, Tokyo, Japan), OCT angiography (RTVue-XR Avanti; Optovue, Software Version 2018.0.0.43, Fremont, CA, USA), measurements of the axial length (AL),corneal curvature, ACD and lens thickness (LT) using ocular biometry (IOL Master 700; Carl Zeiss Meditec, Germany). BCVA was converted into log MAR and spherical equivalent (SE) was used for final analysis. The eyes with the following typical visible features on SS-OCT radial scans centered on disc indicating PS edges as described by Shinohara et al. [13] were considered to have PPS. 1) The posterior sclera in the region of the PPS bending posteriorly with the local radius of curvature less than the radius of curvature of the adjacent scleral regions. 2) The inward protrusion of the sclera with the compressing and thinning choroid at the staphyloma edge. An example of PPS OCT images is shown in Fig. 1.

Inclusion criteria: 24 mm < AL < 30 mm; aged between 18 and 50 years, and IOP < 21 mm Hg. Exclusion criteria: subjects older than 50 years, considering that lenticular changes might affect refractive error and ocular imaging; a history of intraocular or refractive surgery; poor quality of OCT or OCTA images; a history of ocular or systemic diseases that could affect blood flow, including diabetes, hypertension, hematologic disorders. Only one eye was randomly selected to be included in the study when both eyes of a patient were eligible.

### Propensity score matching analysis

The closest eye in the control group to match an eye in the PPS group was selected by the propensity score matching analysis. The program using for propensity score matching was the SPSS software (version 25.0, IBM-SPSS, Chicago, IL). Multivariate logistic regression analysis for each eye was performed to construct propensity scores matching model, where age and AL were used as the concomitant variable. Matching algorithm: 1:1 match, caliper matching with match tolerance of 0.05. Whenever several eyes in the control group could be matched, the eye of nearest propensity score value would be selected as the matched object. At the end of the analysis, 41 eyes from the PPS group and 41 eyes from the control group were matched.

### SS-OCT image acquisition and analysis

Using a 1050-nm wavelength light source, the SS-OCT system was used to provide an A-scan rate of 100000 Hz per second. The depth resolution was 8 um with a lateral resolution of 20um. To correct the amplification factors related to AL, parameters including AL, cornea curvature radius, spherical diopter, and cylindrical diopter were input into the system before image capturing. Three-dimensional cube scan patterns centered on optic disc and macula with a scan size of 6 × 6 mm and 7 × 7 mm respectively were performed to acquire the average peripapillary retinal nerve fiber layer (RNFL) thickness and choroidal thickness (CT) as well as average thicknesses of macular retinal, ganglion cell complex (GCC) and choroidal layers. In general, automatic segmentation by the built-in software was performed to acquire each layer’s thickness, and manual segmentation was used whenever there was an error in judging the borderline of each layer. Retinal thickness (RT) and CT tomography maps of 6 × 6 mm area formed from above three-dimensional imaging data set were centered on macula and used from Early Treatment Diabetic Retinopathy Study grid. As a result, three concentric circles of nine regions were formed, viz., the central circle (0.5 mm radius), inner circle (1.5 mm radius), and an outer circle (3 mm radius). The central and inner region data were used in the final analyses. As for the optic disc region, the average thickness in four quadrants due to the absence of choroid tissue in the optic disc was analyzed.

Additionally, 12 radial scans with a 12 mm scan view centered on the optic disc were obtained to identify the PPS edges and the presence of peripapillary intrachoroidal cavitation (ICC) and retinoschisis. The five-line cross scan centered on the disc with a 6 mm scan length was performed to obtain horizontal and vertical images. The horizontal and vertical scan images passing through the center of the disc were used to measure the horizontal and vertical disc tilt degree. Details are described below.

### Measurements of optic disc area, parapapillary atrophy (PPA) area, rotation, and tilt

**Fig. 2:**
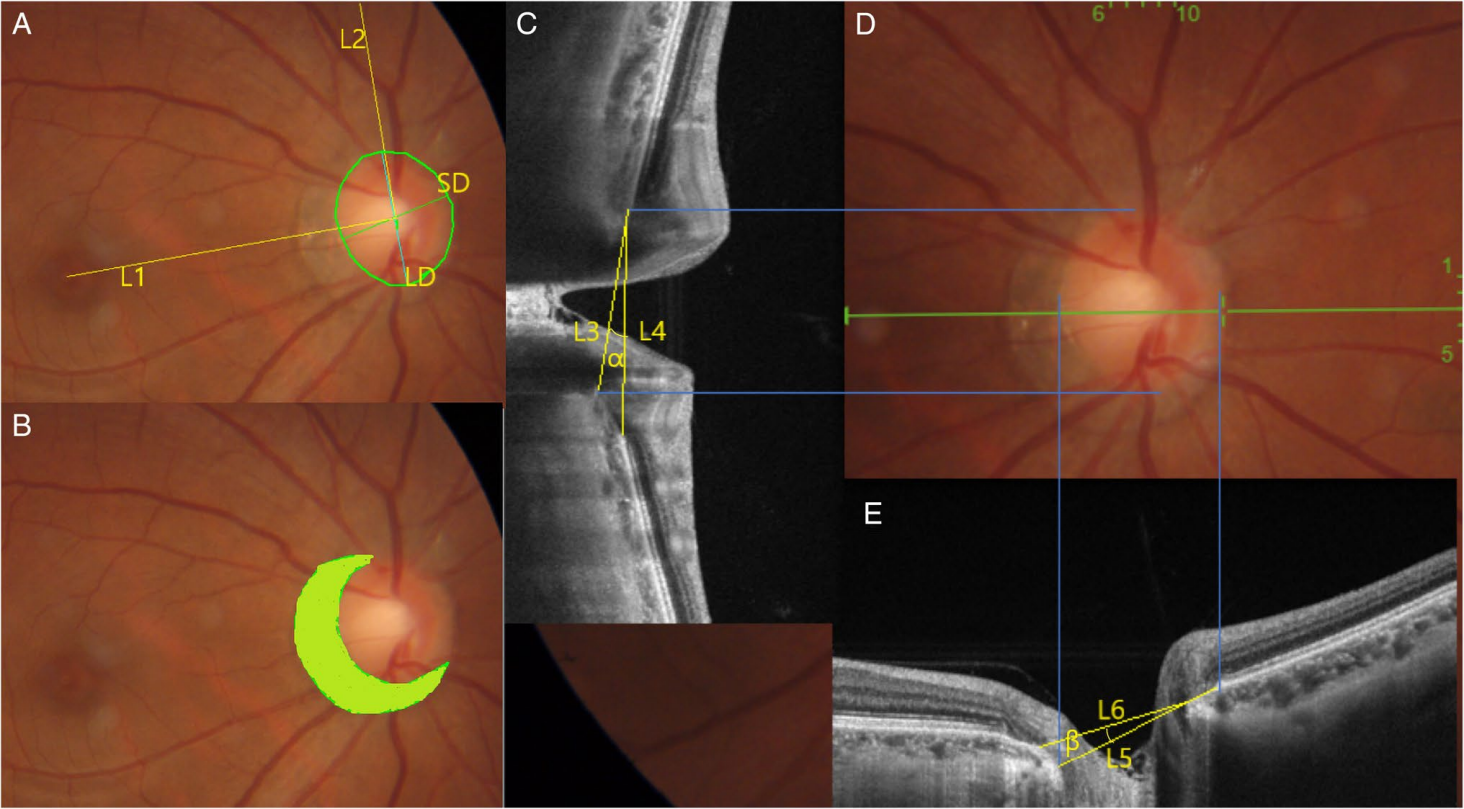
Measurement of optic disc tilt, rotation, and PPA area. (**A**) The green lines represent the SD and the LD of the optic disc. The tilt ratio was defined as the ratio of the LD to the SD. The degree of rotation was measured between the LD and the vertical meridian (L2)), identified as a vertical line 90°from the horizontal line (L1) connecting the fovea and the center of the optic disc. (**B**) The PPA margins were outlined manually. (**C-E**) Disc tilt degree was determined from horizontal and vertical OCT scans passing through optic disc center. (**D**). The clinical disc margins on the disc photograph were marked on the corresponding OCT scans, where they met the border tissue or BM (C, E; blue line). The ONH plane refers to a line connecting two points, marking the clinical disc margin on OCT scan (C, E; L3, L5), while the reference plane refers to another line connecting Bruch's membrane opening (BMO) on the corresponding OCT scan (C, E; L4, L6)

Fundus photographs centered on the macula and optic disc were captured using standardized system settings and then imported as a TIFF image file. Optic disc area, PPA area, tilt, and rotation were measured using image analysis software (Image-Pro Plus 6.0.) by two independent retinal ophthalmologists (FN and LZ) (Fig. 2). The clinical disc and PPA (chorioretinal atrophy region characterized by visible large choroidal vessels and sclera) margins on disc photograph were marked based on the experience of ophthalmologists, where the area was determined as the total number of pixels. The definitions and measurements of optic disc rotation and tilt described previously were adopted [14, 15]. Specifically, disc rotation was identified by the deviation of the longest diameter (LD) of the disc from the reference line, 90°from a horizontal line connecting the fovea and the center of the disc. The degree of rotation was regarded as the angle between the LD of the disc and the reference line. When the degree of rotation was greater than 15°, the disc was considered rotated. A positive value suggested an inferotemporal rotation, and a negative value suggested a supranasal rotation. Disc tilt was determined by tilt ratio, defined as the ratio between the LD and the disc’s shortest diameter (SD). When the tilt ratio was greater than 1.3, the disc was considered tilted. Disc tilt was also identified from SS-OCT horizontal and vertical scans, and its measurement has been introduced [16, 17]. The horizontal scan is aligned with an imaginary line connecting the fovea and the center of the disc. The clinical disc margins on the disc photograph were marked on the corresponding OCT scans, where they met the border tissue or Bruch's membrane (BM). The optic nerve head (ONH) plane refers to a line connecting these two points, marking the clinical disc margin on OCT scan. In contrast, the reference plane refers to another line connecting Bruch’s membrane opening (BMO) on the corresponding OCT scan. Disc tilt degree was defined as the angle between the above two lines. Similarly, the degree of vertical tilt was measured from a vertical OCT scan in the same way described above. A positive value suggested an inferior tilt; conversely, a negative value suggested a superior tilt. The absolute value of the degree of rotation was used in the current study when performing statistical analysis, which is also applied to analyze disc tilt degree.

### OCT angiography image acquisition and analysis

**Fig. 3:**
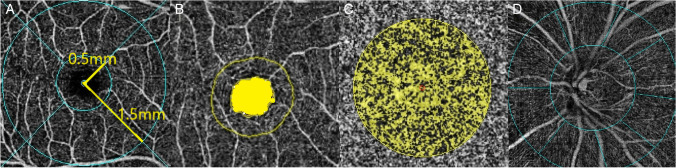
Measurement of vessel parameters. (**A**) Fovea region (inner circle of 0.5 mm radius) and parafovea region (1 mm width annulus, outer circle of 1.5 mm). (**B**) FAZ area. (**C**) CC flow area (circle of 1 mm radius centered on fovea). (**D**) Peripapillary region (0.75 mm width elliptical annulus centered on disc, extending outward from disc margins to the RPC segment)

Using an 840-nm wavelength light source, the OCT angiography system provided an A-scan rate of 70000 Hz per second. Cube scan patterns centered on the optic disc and macula with scan size of 4.5 × 4.5 mm and 6 × 6 mm were performed. These scans acquired perfusion density of radial peripapillary capillary (RPC), macula superficial and deep retinal capillary, foveal avascular zone (FAZ) area, choriocapillaris (CC) flow area (circle with a 1 mm radius centered on fovea). All the above vessel parameters were outcomes obtained automatically. Perfusion density of RPC was measured in region centered on disc, a 750-um width elliptical annulus, extending outward from disc margins to the RPC segment. Similarly to RT and CT maps, superficial and deep retinal capillary were measured in two concentric circles of five regions, viz., the inner circle (0.5 mm radius, fovea), outer circle (1.5 mm radius, parafovea). Figure 3 illustrates the measurements of the vessel parameters detailed.

### Statistical analysis

All statistical analyses were conducted with SPSS ver. 25.0 software (IBM-SPSS Inc., Chicago, IL). The statistical normal distribution was evaluated with Kolmogorov–Smirnov normality test. Whereas Mann–Whitney *U* test, independent *t*-test, and χ^2^ test were applied to assess the differences between groups. Spearman’s correlation analysis was used to evaluate the association between the ocular parameters and the degree of rotation. Univariate and multivariate logistic regression analyses were conducted to determine the factors associated with PPS. The variables with *P* value < 0.1 in the univariate analysis were included in the multivariate model. Significant *P* value statistically was set at 0.05.

## Results

### General and anterior segment characteristics

As shown in Table 1, age, sex, CCT, AL, and cornea curvature were similar between the PPS and control groups. The PPS group exhibited higher IOP than control group (16.59 ± 2.88 mmHg vs 14.53 ± 2.45 mmHg, *P* = 0.002). However, even though AL was matched, and no significant difference was observed in cornea curvature among the groups, the SE of eyes with PPS (− 11.52 ± 3.22D) was higher than eyes in control group (-9.88 ± 2.20D) (*P* = 0.009). As was expected, the BCVA of PPS group was worse compared with control group (*P* < 0.001). Additionally, the PPS group showed a shallower ACD than the control group (3.61 ± 0.24 mm vs 3.77 ± 0.24 mm, *P* = 0.004). Interestingly, although there was no statistical difference in LT between the two groups, the mean value of LT in the PPS group was thicker than the control group (3.80 ± 0.26 mm vs. 3.68 ± 0.19 mm, *P* = 0.101).

**Table 1:** Subject’s general characteristics and anterior segment variables of the PPS and the control group eyes matched according to age and AL

Variables	PPS group *n* = 41	Control group *n* = 41	P value
Age, y	28.52 ± 8.05	28.06 ± 7.31	0.818*
Sex, male/female	8/33	14/27	0.212 †
IOP, mmHg	16.59 ± 2.88	14.53 ± 2.45	0.002*
CCT, µm	523.47 ± 35.14	515.00 ± 30.60	0.281*
SE, D	− 11.52 ± 3.22	− 9.88 ± 2.20	0.009 *
BCVA, logMAR	0.10 ± 0.10	0.01 ± 0.02	** < 0.001‡**
AL, mm	28.01 ± 0.87	28.02 ± 0.88	0.970 ‡
Cornea curvature, D	43.39 ± 1.52	42.86 ± 1.22	0.086 *
ACD, mm	3.61 ± 0.24	3.77 ± 0.24	0.004 *
LT, mm	3.80 ± 0.26	3.68 ± 0.19	0.101 ‡

*IOP* intraocular pressure, *CCT* central corneal thickness, *SE* spherical equivalent, *BCVA* best-corrected visual acuity, *AL* axial length, *ACD* anterior chamber depth, *LT* lens thickness. *RPC* radial peripapillary capillary. Factors with statistical significance are shown in boldface. *Independent *t*-test, † *χ*2 test, ‡ Mann–Whitney *U* test

### Optic disc, RNFL, and GCC characteristics

Variables related to disc, RNFL, and GCC were compared between the groups (Table 2). Larger disc and PPA were observed in the PPS group (*P* < 0.001 and *P* < 0.001, respectively). PPS group exhibited greater disc rotation (41.17 ± 29.53°vs. 14.78 ± 16.09°) than the control group (*P* < 0.001). Peripapillary ICC was detected in 1 eye of 41 eyes with PPS, but no one in the control group. Peripapillary retinoschisis was found in 21 eyes in the PPS group, while one eye was in the control group. No significant differences in disc tilt ratio, horizontal tilt angle, vertical tilt angle were identified. As for the peripapillary RNFL, no significant differences were observed in nasal, temporal, and arithmetic mean of four quadrants RNFL thicknesses between the groups (*P* = 0.099, *P* = 0.640, and *P* = 0.073, respectively). In contrast, the superior (112.05 ± 31.92 µm vs. 124.85 ± 16.86 µm) and inferior (118.90 ± 22.48 µm vs. 129.10 ± 21.56 µm) RNFL thickness were significantly different between the groups (*P* = 0.026 and *P* = 0.039, respectively). Similarly, superior (101.05 ± 6.95 µm vs. 106.22 ± 8.15 µm), inferior (102.83 ± 7.79 µm vs. 107.66 ± 7.72 µm) and average (101.83 ± 6.99 µm vs. 106.95 ± 7.64 µm) GCC thickness was also significantly different between PPS and control eyes with myopia (*P* = 0.001, *P* = 0.007, and *P* = 0.001, respectively). Details are shown in Table 2.

**Table 2:** Optic disc, RNFL, and GCC characteristics of the PPS and the control group eyes matched according to age and AL

Variables	PPS group, *n* = 41	Control group, *n* = 41	*P* value
Disc area, pixel	66,310.39 ± 18,826.37	49,418.32 ± 8706.13	< 0.001*
PPA area, pixel	54,235.15 ± 42,044.25	23,490.22 ± 12,978.71	** < 0.001‡**
Type of PPA			
Annular	10(24.4%)	0	
Crescent	29(70.7%)	40(97.6%)	
Absence	2(4.9%)	1(2.4%)	
Tilt ratio	1.23 ± 0.13	1.26 ± 0.12	0.240*
Degree of rotation, °	41.17 ± 29.53	14.78 ± 16.09	** < 0.001‡**
Horizontal tilt angle, °	12.65 ± 8.08	10.21 ± 5.40	0.112*
Vertical tilt angle, °	5.30 ± 5.00	3.87 ± 4.64	0.184‡
Peripapillary ICC			
Presence/absence	1/40	0/41	** < 0.001†**
Peripapillary retinoschisis			
Presence/absence	21/20	1/40	** < 0.001†**
RNFL thickness, µm			
Superior	112.05 ± 31.92	124.85 ± 16.86	0.026*
Nasal	67.73 ± 19.29	61.49 ± 14.15	0.099*
Inferior	118.90 ± 22.48	129.10 ± 21.56	0.039*
Temporal	94.54 ± 17.58	96.41 ± 18.59	0.640*
Average RNFL thickness	98.30 ± 14.03	103.03 ± 9.06	0.073*
GCC thickness, µm			
Superior	101.05 ± 6.95	106.22 ± 8.15	**0.001‡**
Inferior	102.83 ± 7.79	107.66 ± 7.72	**0.007‡**
Average GCC thickness	101.83 ± 6.99	106.95 ± 7.64	**0.001‡**

*PPA* parapapillary atrophy, *ICC* intrachoroidal cavitation, *RNFL* retinal nerve fiber layer, *GCC* ganglion cell complex. *RPC* radial peripapillary capillary. Factors with statistical significance are shown in boldface. *Independent *t*-test, † *χ*2 test, ^‡^ Mann–Whitney *U* test

### Retinal and choroidal thickness in macular and optic disc region

Compared with the control group, inner region RT, macular and peripapillary CT was dramatically thinner in the PPS group. However, no significant difference in central region RT were identified between groups. Details are displayed in Table 3.

**Table 3:** Regional retinal and choroidal thickness of the PPS group and the control group eyes matched according to age and AL

Variables	PPS group, *n* = 41	Control group, *n* = 41	*P* value
Central region RT, µm	240.93 ± 17.77	239.98 ± 13.07	0.783*
Inner regions RT, µm			
Superior	295.54 ± 12.36	304.73 ± 14.38	0.003*
Nasal	295.41 ± 12.58	307.29 ± 13.95	< 0.001*
Inferior	290.98 ± 12.98	301.30 ± 13.81	**0.001‡**
Temporal, um	283.68 ± 11.85	290.28 ± 13.18	**0.021‡**
Central region CT, µm	125.98 ± 50.47	179.22 ± 61.90	< 0.001*
Inner regions CT, µm			
Superior	137.22 ± 50.57	190.22 ± 62.16	**0.001‡**
Nasal	94.41 ± 35.37	153.63 ± 49.45	** < 0.001‡**
Inferior	125.59 ± 47.69	183.49 ± 65.05	< 0.001*
Temporal	152.20 ± 57.44	201.63 ± 65.45	< 0.001*
Peripapillary CT, µm			
Superior	87.71 ± 29.88	139.07 ± 41.98	** < 0.001‡**
Nasal	79.20 ± 39.66	128.85 ± 37.37	< 0.001*
Inferior	62.49 ± 34.98	101.24 ± 32.17	< 0.001*
Temporal	58.46 ± 25.88	95.51 ± 35.23	** < 0.001‡**
Average Peripapillary CT	70.46 ± 27.57	116.17 ± 34.17	< 0.001*

*RT* retinal thickness, *CT* choroidal thickness. *RPC* radial peripapillary capillary. Factors with statistical significance are shown in boldface. * Independent *t*-test, ‡ Mann–Whitney *U* test

### Microvasculature characteristics measured by OCT angiography

The vessel parameters obtained by OCT angiography were compared between the groups (Table 4). No significant differences were observed in macular superficial vessel density and FAZ area. Conversely, for the macular deep microvasculature, the vessel density was consistently higher in the PPS group versus the control group for superior (53.14 ± 4.35% vs. 50.54 ± 4.69%, *P* = 0.011), nasal (55.47 ± 4.42% vs. 53.49 ± 4.42%, *P* = 0.045), and inferior (51.54 ± 5.99% vs. 48.68 ± 6.74%, *P* = 0.045) quadrants whereas was not significantly different in the fovea, parafovea, and temporal quadrant (*P* = 0.708, *P* = 0.053, and *P* = 0.898, respectively). For the RPC, the vessel density was consistently lower in the PPS group versus the control group for superior (47.63 ± 7.81% vs. 52.73 ± 4.08%, *P* = 0.001), inferior (49.24 ± 6.59% vs. 53.76 ± 4.28%, *P* < 0.001), temporal (50.66 ± 6.35% vs. 54.83 ± 6.76%, *P* < 0.001) quadrants, and the mean of four quadrants (47.85 ± 4.92% vs. 51.30 ± 3.51%, *P* < 0.001) whereas was not significantly different in nasal quadrants (*P* = 0.255). CC flow area was significantly decreased in the PPS group versus the control group (2.02 ± 0.10mm^2^ vs. 2.07 ± 0.13mm^2^, *P* = 0.034).

**Table 4:** Vessel parameters using OCT angiography of the PPS group and the control group eyes matched according to age and AL

Variables	PPS group, *n* = 41	Control group, *n* = 41	*P* value
Superficial vascular density, %			
Fovea	23.46 ± 7.07	22.33 ± 6.30	0.447*
Parafovea	49.29 ± 4.74	49.24 ± 4.74	0.948‡
Superior	51.59 ± 4.31	50.44 ± 5.20	0.478‡
Nasal	47.08 ± 7.36	47.99 ± 5.62	0.959‡
Inferior	48.43 ± 6.79	49.41 ± 5.30	0.930‡
Temporal	50.04 ± 5.63	49.11 ± 5.11	0.241‡
Deep vascular density, %			
Fovea	38.51 ± 7.08	39.08 ± 6.59	0.708*
Parafovea	53.59 ± 4.26	51.71 ± 4.38	0.053*
Superior	53.14 ± 4.35	50.54 ± 4.69	0.011*
Nasal	55.47 ± 4.42	53.49 ± 4.42	0.045*
Inferior	51.54 ± 5.99	48.68 ± 6.74	0.045*
Temporal	54.23 ± 4.24	54.11 ± 4.39	0.898*
RPC density, %			
Superior	47.63 ± 7.81	52.73 ± 4.08	**0.001‡**
Nasal	44.76 ± 4.54	45.88 ± 4.32	0.255*
Inferior	49.24 ± 6.59	53.76 ± 4.28	< 0.001*
Temporal	50.66 ± 6.35	54.83 ± 6.76	** < 0.001‡**
Average RPC density	47.85 ± 4.92	51.30 ± 3.51	< 0.001*
FAZ area, mm^2^	0.22 ± 0.10	0.24 ± 0.09	0.478‡
CC flow area, mm^2^	2.02 ± 0.10	2.07 ± 0.13	0.034*

*RPC* radial peripapillary capillary, *FAZ* foveal avascular zone, *CC* Choriocapillaris. Factors with statistical significance are shown in boldface. * Independent *t*-test, ‡ Mann–Whitney *U* test

### Independent factors associated with PPS, the correlation between the ocular factors and disc rotation

**Fig. 4:**
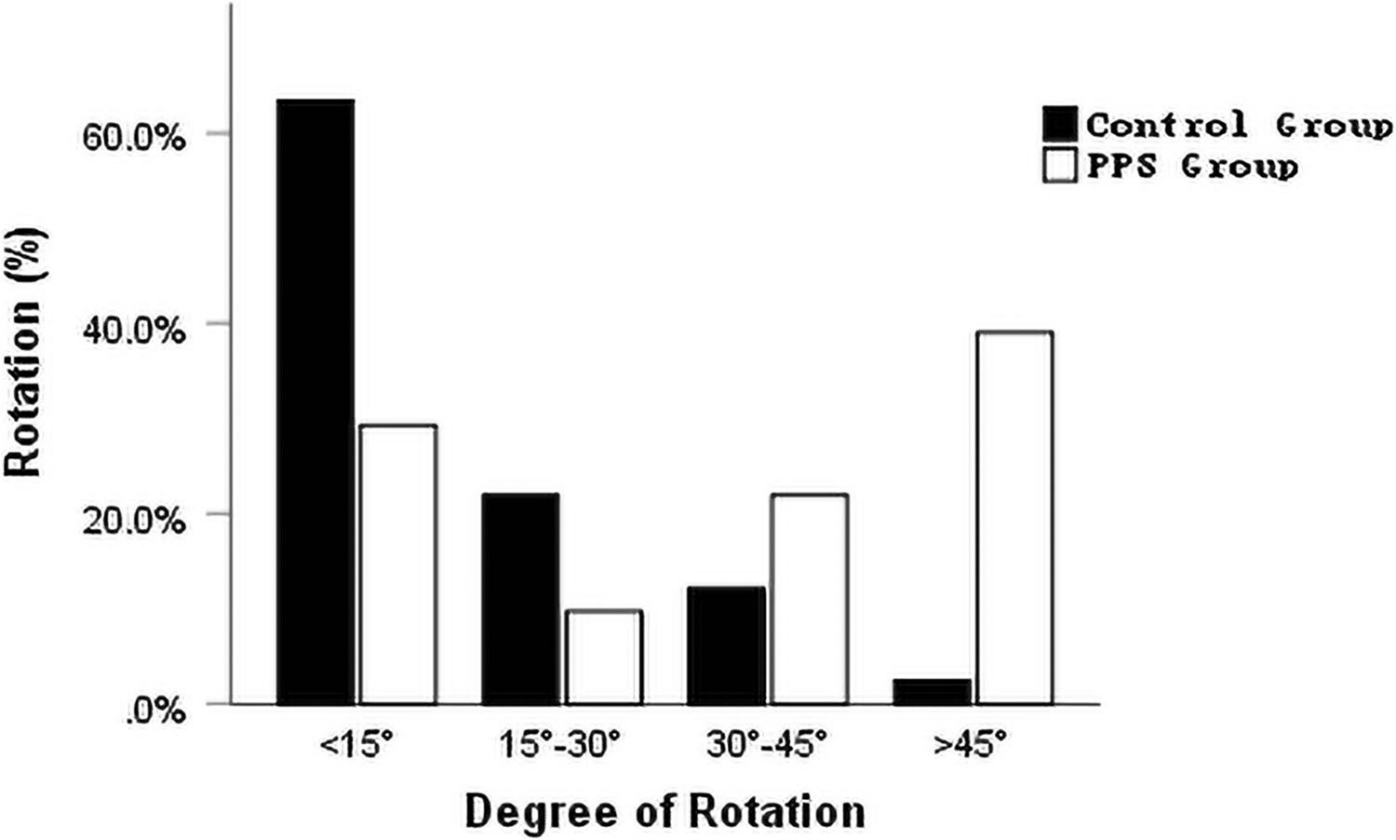
Histogram exhibiting the distribution of the disc rotation. The degree of rotation was distributed less than 15°, between 15°and 30°, between 30°and 45° and more than 45°for PPS and normal controls group

**Fig. 5:**
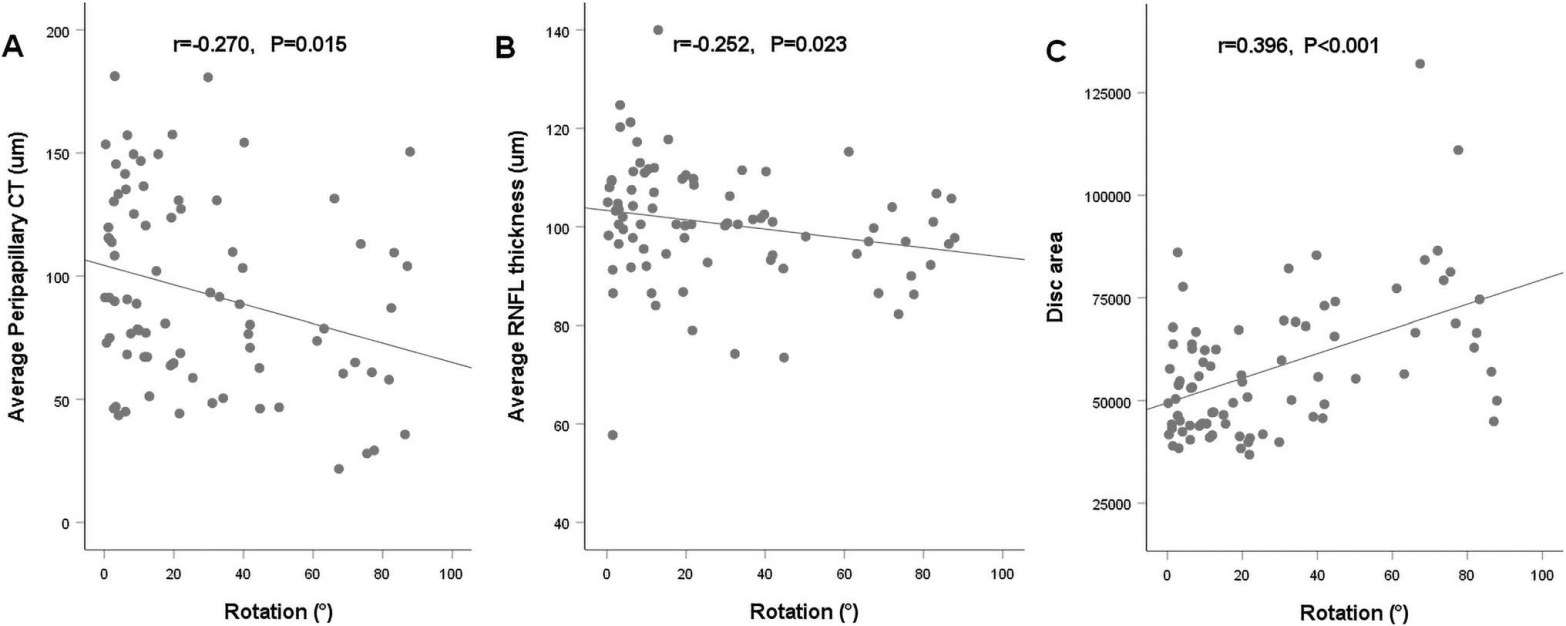
Scatterplots of the correlation between the degree of rotation and (**A**) average peripapillary CT, (**B**) average RNFL thickness, and (**C**) disc area

Association between various ocular parameters and PPS presence were analyzed in the PPS and the control group eyes matched according to age and AL using a logistic regression model (Table 5). Univariate regression analysis demonstrated that the IOP (*P* = 0.004), SE (*P* = 0.013), ACD (*P* = 0.006), average peripapillary CT (*P* < 0.001), average RNFL thickness (*P* = 0.081), disc area (*P* < 0.001), PPA area (*P* < 0.001), and degree of rotation (*P* < 0.001) were factors related to PPS. Multivariate logistic regression analyses identified that IOP (*P* = 0.046), average peripapillary CT (*P* = 0.009), and degree of rotation (*P* = 0.003) were independent factors associated with the presence of PPS. The distribution of disc rotation in eyes matched according to age and AL are shown in Fig. 4. The relationship between the degree of rotation and ocular factors are displayed in Fig. 5. The degree of rotation was negatively correlated with average peripapillary CT (*r* =  − 0.270, *P* = 0.015) and average RNFL thickness (*r* =  − 0.252, *P* = 0.023) and positively correlated with disc area (*r* = 0.396, *P* < 0.001).

**Table 5:** Univariate and multivariate logistic regression analyses of PPS

	Univariate		Multivariate	
	B (95% CI)	*P* value	B (95% CI)	*P* value
Age, y	0.012 (0.953–1.075)	0.690		
AL, mm	− 0.016 (0.569–1.623)	0.949		
IOP, mmHg	0.292 (1.099–1.634)	0.004	**0.397 (1.007–2.198)**	**0.046**
SE, D	− **0.227 (0.666–0.953)**	**0.013**	0.017 (0.719–1.440)	0.922
ACD, mm	− **2.690 (0.010–0.468)**	**0.006**	− 2.447 (0.002–3.550)	0.197
Average peripapillary CT, µm	− **0.044 (0.939–0.976)**	** < 0.001**	− **0.059 (0.902–0.985)**	**0.009**
Average RNFL thickness, µm	− **0.036 (0.927–1.004)**	**0.081**	0.019 (0.922–1.126)	0.710
Optic disc area, pixel	** < 0.001 (1.000–1.000)**	** < 0.001**	< 0.001 (1.000–1.000)	0.718
PPA area, pixel	** < 0.001 (1.000–1.000)**	** < 0.001**	< 0.001 (1.000–1.000)	0.709
Rotation,°	**0.048 (1.024–1.075)**	** < 0.001**	**0.074 (1.025–1.131)**	**0.003**
Tilt ratio	− 2.162 (0.003–4.178)	0.238		
Horizontal tilt angle, °	0.053 (0.987–1.127)	0.116		
Vertical tilt angle, °	0.063 (0.971–1.168)	0.184		
Average RPC density, %	− **0.199 (0.724–0.928)**	**0.002**	− 0.253 (0.590–1.021)	0.070

*AL* axial length, *IOP* intraocular pressure, *SE* spherical equivalent, *ACD* anterior chamber depth, *CT* choroidal thickness, *RNFL* retinal nerve fiber layer, *PPA* parapapillary atrophy, *RPC* radial peripapillary capillary. Factors with statistical significance are shown in boldface. *B* unstandardized coefficient, *CI*, confidence interval

## Discussion

In the present study, the eyes with PPS exhibited a higher degree of myopic refractive error with no significant difference in AL and corneal curvature between the groups. Furthermore, the eyes with PPS had shallower ACD with thicker LT, though the difference of LT was statistically insignificant possibly due to the small sample size. Our results were in-concurrence with the previous studies report linking PS with the higher myopic refractive error and anterior segments anatomical abnormalities [3], though it was not clear which type of PS contributed to this, we speculate the wide, macular staphyloma might have resulted for the following reasons: 1) this staphyloma type accounts for approximately three-quarters of all staphyloma [1, 2]; and 2) it involving the temporal side of the optic disc is characterized by a small disc with large tilt ratio, etc. [6], thus consistent with our previous findings [3]. Hence, either wide, macular staphyloma or PPS might have an association with anterior segments anatomical abnormalities and myopic SE refractive error. To the best of our knowledge, this phenomenon has not been previously reported and the mechanisms to explain anterior segments anatomical abnormalities remains unclear. Liu and colleagues [18] reported that eyes with more apparent PS visible on fundus imaging had significantly shorter AL than the eyes without fundus pigmentary changes. As PS angle size (an index of describing the edge of the PS) increased, AL significantly decreased. This suggests that the AL may become shorter as the PS develops. Inward scleral forces at PS edges may push the posterior globe anteriorly, while forming staphyloma angles and a visible staphylomatous outpouching. In view of the findings of Liu et al., the inward scleral forces may also cause lens thickening, anterior chamber shallowing, and shortening of vitreous chamber. The increase of refractive error in myopia with anterior segments anatomical abnormalities of the eye with PS or PPS can be explained by this mechanism. Of course, some changes in the anterior segments can also be the reason, such as loose suspensory ligament, the changes of lens structure and/or refractive index. However, longitudinal studies are still needed to validate these observations. Our data revealed that the eye with PPS were characterized by 1) a large and rotated disc area, 2) thinning of GCC, superior or/and inferior peripapillary RNFL, 3) decrease in superior or/and inferior RPC (Tables 2 and 4), and 4) higher IOP (Table 1). Moreover, large optic disc size usually has a greater cup-to-disc ratio, which is extremely like glaucomatous optic disc, thus accurate diagnosis is challenging. Given that the prevalence of PPS in myopic patients with glaucoma was much higher than that in myopic people [6], it is reasonable to suspect that PPS may be a risk factor for glaucoma combined with our results. It should be pointed out that the IOP in present study was measured by non-contact tonometer other than Goldmann applanation tonometry, thus the possibility of big error cannot be excluded. Additionally, studies on high myopic patients suggested that the glaucoma-like visual field defects were detected more frequently in eyes with peripapillary ICC than in eyes without ICC (64.3% vs.19.5%) [19]. The prevalence of ICC in eyes with PPS was 52.5% in reports by Shinohara et al. [13], which was significantly higher than findings of our study (2.4%) because of our young subjects. Therefore, understanding the microvascular, structural, and visual field characteristics of optic discs in myopic eyes with PPS and the impact of PPS on ocular imaging is vital for accurate diagnosis of glaucoma as well as the prompt and reasonable treatment. Nonetheless, ophthalmologists would be able to accurately diagnose glaucoma and develop therapeutic plans based on the longitudinal follow-up in myopic eyes with PPS. Disc area of eyes with PPS was significantly larger than the non-staphylomatous eyes. This anomaly might be explained by 1) the stress exerted due to the intraocular pressure pushing disc posteriorly, and 2) by LaPlace’s law, that the expansion of shell in peripapillary region may create higher stress that enlarge disc extensively, which results in annular PPA and untilted optic disc. Recent studies by Kim et al. [20] have also confirmed this hypothesis of the disc being pulled toward the deepest point (DPE, defined as the deepest interface between Bruch's membrane and the choroid) of the eyeball, that determines a myopic disc configuration. Moreover, corresponding with our hypothesis, the DPE of eyeball with PPS was also located at the optic disc, in other words, the optic disc was pulled posteriorly. In general, the expansion of the scleral canal and the lamina cribrosa occurs with the enlargement of the optic disc. Therefore, in case of lamina cribrosa thinning, trans-laminar cribrosa pressure difference increases, which further strongly associates PPS with increased susceptibility to glaucoma. Consistent with Nie et al., macular CC flow area in eyes with PPS also decreased [3]. The compensatory increase of deep retinal flow blood occurring in parafovea superior, nasal, inferior, namely region near the PPS was observed in our study, however, no such difference was observed in study by Nie et al. [3]. This finding indicates that the decrease in choroidal blood flow precedes the changes in retinal perfusion. Therefore, choroid appears to be the primary structure originally affected in the process of staphyloma formation. Furthermore, corresponding with our previous study [3], CT was a signal of staphyloma formation, however the difference of disc rotation instead of disc tilt was found to be another a signal of staphyloma formation. These pieces of evidence strongly suggest that the different types of staphyloma determine the variable disc configuration. Interestingly, inner region retinal thinning occurs despite compensatory increases of deep retinal blood flow at the parafovea. The remarkable differences in BCVA (0.10 ± 0.10 vs 0.01 ± 0.02) implies that the functional decompensation has already appeared in PPS group, though no differences were observed in macular central region RT, fovea vascular density and FAZ area among the studied groups. Disc rotation is a significant signal of PPS formation, therefore it was vital to perform a linear correlation analysis between the disc rotation and the ocular factors. Our data shows that disc rotation was closely correlated with the thinning choroid and RNFL, and larger disc. However, previous studies have reported that the myopic patients with primary open-angle glaucoma showed PS involvement in the disc, enlarges the disc size extensively with the less disc tilt or rotation [6, 15]. We suspect that optic discs in the present study were not really rotating, however the asymmetry in the enlargement of the optic disc occurred with asymmetry of the expansion of sclera in peripapillary region. For example, when the sclera stretched more horizontally than vertically, the optic disc expanded transversely than longitudinally, leading it to appear as a transversely elliptical optic disc.

We acknowledge that several limitations exist in the present study. The major limitation was the small sample size as it was not easy to enroll a myopic patient with PPS owing to its low prevalence, particularly in young subjects. Nevertheless, there is compelling evidence showing that age is one of essential risk factor for PS [21, 22]. Secondly, the cross-sectional design and single center background did not explain the causal association between PPS with the microvascular and structural changes in myopic eyes.

In conclusion, peripapillary and macular choroidal perfusion significantly reduces with compensatory increase in macular deep retinal blood flow of myopic eyes with PPS. Furthermore, PPS is associated with exacerbation of myopia and anatomical alterations, including eye anterior segment as well as posterior segment. Overall, PPS may lead to various mechanical changes in the surrounding sclera, retina and choroid, which may explain the different phenotypes, and our findings may be helpful in giving new insights into differentiating glaucoma from myopia.

## Supplementary information

Below is the link to the electronic supplementary material.

Supplementary file1 (XLSX 50 KB)

## Data Availability

The original contributions presented in the study are included in the article/supplementary material, further inquiries can be directed to the corresponding author.
